# Prevention and treatment of neonatal hypothermia through an implementation science study in Jaltenango Chiapas, Mexico

**DOI:** 10.7189/jogh.15.04180

**Published:** 2025-06-02

**Authors:** Alejandro Frade Garcia, Lucila Servitje Azcarraga, María Azucena Espinosa Olivas, Laila Zulema García Ulloa, Rodrigo Garcia Santisteban, Anne Hansen

**Affiliations:** 1 Boston Children’s Hospital, Boston, Massachusetts, USA; 2 Harvard Medical School, Boston, Massachusetts, USA; 3 Universidad Panamericana, Mexico City, Mexico; 4 Hospital Basico Comunitario Angel Albino Corzo, Chiapas, Mexico

## Abstract

**Background:**

Neonatal hypothermia is preventable but common, contributing to neonatal morbidity and mortality especially in low resource settings. Kangaroo Mother Care (KMC) is the preferred method to prevent hypothermia, but relying on it as a continuous heat source is challenging. This study introduced a novel Infant Warmer to complement KMC in a low resource community hospital through an implementation science approach.

**Methods:**

We conducted a prospective, interventional cohort study in Jaltenango Chiapas, Mexico from January 2022 to November 2022. Our intervention was 1) an educational programme about the importance of euthermia and optimal thermoregulatory practices including KMC, and 2) provision of an Infant Warmer designed for low-resource settings with training of health care providers. Our hypothesis was that neonatal hypothermia rates would decrease after our intervention. Our aims were to reduce rates of hypothermia and increase knowledge and confidence regarding neonatal thermoregulation. The study had three phases: Pre-Intervention (January to May), Intervention, and Warmer Use (June to November). We collected clinical data during the Pre-Intervention and Warmer Use Phases, including temperature on admission and six hours later called ‘Follow-Up’. At three-time points we conducted surveys of health care providers regarding their knowledge of hypothermia, confidence in keeping babies warm, and attitudes regarding the Warmer. We also conducted a parent survey.

**Results:**

We studied 372 newborns. Comparing Pre-Intervention to Warmer Use Phases, rates of hypothermia decreased from 62 to 27% on admission and 59 to 11% on Follow-Up. The mean admission and Follow-Up temperatures increased by 0.06°C (C) and 0.23°C (*P* = 0.003) respectively. Healthcare providers' knowledge of hypothermia and confidence in keeping newborns warm also improved throughout the study.

**Conclusions:**

We found high baseline rates of neonatal hypothermia in this low resourced hospital. We successfully lowered hypothermia rates by providing appropriate equipment to complement KMC and improved knowledge of hypothermia through education interventions.

Neonatal hypothermia is a common, preventable cause of morbidity and mortality worldwide, primarily affecting those born low birth weight (LBW) and premature [1–3]. Hypothermia in newborns has a negative effect on multiple body systems, including cardiac, pulmonary, immune, and metabolic. It also stunts normal growth and development [4–6] as calories are shunted from somatic growth to heat generation. Avoiding hypothermia is a basic requirement to maximise newborn survival and outcome [7–9].

Globally, newborns in all income settings suffer from hypothermia, with overall reported in-hospital rates ranging from 32 to 85% [7,10]. Yet it disproportionally affects newborns in low- and middle-income countries including those in warmer climates [11]. Despite thermoregulation’s widely accepted role as a fundamental element in newborn care, rates of neonatal hypothermia remain high, especially in low-resourced areas [11–13].

Kangaroo Mother Care (KMC) is the recommended approach to prevent and treat neonatal hypothermia in resource-limited settings [8]. It consists of skin-to-skin contact between mother and newborn. According to a meta-analysis, LBW newborns who underwent continuous KMC compared to conventional care had a 36% lower mortality rate (relative risk (RR) = 0.64; 95% confidence interval (CI) = 0.46, 0.89), and decreased risk of hypothermia, hypoglycaemia, sepsis, and hospital readmission [14]. However, hypothermia prevention relies on this skin-to-skin contact being continuous, which is difficult for families to sustain. This results in gaps in KMC provision. Only 6.5% of mothers stated it is feasible to provide KMC for at least 12 hours a day in one study [15], and it was provided an average of less than three hours per day for eligible newborns three to seven days after birth in another study [16]. Thus, an additional external heat source is needed to supplement KMC for at-risk newborns. In high resourced settings, incubators and radiant warmers serve this purpose; unfortunately, low resourced areas often lack these devices. Even if they are available, there may not be a reliable electricity supply and the capability to properly use and maintain this expensive equipment.

The Dream Warmer was developed as a ‘frugal technology’ [17] to address the challenges of preventing and treating neonatal hypothermia in resource-limited settings. This device is safe, effective, affordable, reusable, and operates without electricity [18–20]. Essentially, it is a mattress composed of a phase change wax material that maintains a temperature of 37°C (C) for up to six hours after being melted. The Dream Warmer can be used as an external heat source when the mother is unavailable to provide KMC, or as a supplement to KMC when it provides insufficient heat.

The Dream Warmer was studied in three clinical trials [18–20] in Rwanda, which together show that in 92% of 1074 uses, it prevented or treated neonatal hypothermia with no adverse events. We then conducted two implementation science studies, in Rwanda and Malawi [21,22] with similar efficacy and safety results and positive reception by health care workers and parents. However, the Dream Warmer has not been assessed outside of the region of sub-Saharan Africa. In an effort to understand its effect, uptake and reception in a Central American context, we collaborated with health care providers at a rural community hospital in the region of Mexico with the highest poverty rate.

Although Mexico is classified as a middle-income country by the World Bank, there are significant variations in health care and resources, with some hospitals and health centres being severely under-resourced [23,24]. Chiapas is a state in Southern Mexico with the country's highest poverty rate (67.4%) and extreme poverty rate (28.2%) [25]. There is a community hospital in the remote community of Jaltenango Chiapas, 90 miles away from the nearest referral hospital with advanced neonatal care facilities in the city of Tuxtla Gutierrez. Due to the absence of medical transportation, sick newborns are frequently managed at the hospital for extended periods before being transferred to a higher level of care. Jaltenango hospital provides services to a population of 70 000 people, including many migrants from Central and South America. It averages approximately 500 deliveries annually and is the primary referral center for ten primary health clinics with a delivery service in the rural communities, and for families who choose home birth. We chose this specific site because the principal investigator had a professional connection to the local providers who reported high rates of neonatal hypothermia and sought a context appropriate solution with training and education. In response to this request, we studied the introduction of the Dream Warmer using an implementation science approach.

While clinical trials focus on testing the safety and effectiveness of interventions, implementation science identifies the barriers and facilitators to the uptake of evidence-based clinical innovations, and how they are adopted and sustained in real-world settings [26]. Our objective was to evaluate the impact of introducing an infant warming device for the prevention and treatment of neonatal hypothermia. We focused on assessing the device's effectiveness by evaluating clinical outcomes, while also examining its uptake and acceptability via survey data.

## METHODS

### Study design

We conducted a prospective, interventional cohort study from January 2022 to November 2022 at the Jaltenango, Chiapas community hospital. In this implementation science study, we employed a Type 1 Hybrid design to evaluate the effectiveness of our intervention and assess the process of the implementation [27].

Our intervention was comprised of:

1) an educational programme about the importance of euthermia and optimal thermoregulatory practices including KMC, and

2) the provision of an Infant Warmer designed for low-resource settings with training of health care providers.

Our hypothesis was that neonatal hypothermia rates would decrease after our intervention. Our aims were to reduce hypothermia and increase knowledge and confidence regarding neonatal thermoregulation.

We based our study on the Reach, Effectiveness, Adoption, Implementation, and Maintenance (RE-AIM) framework [28], a widely used model in implementation science, that aims to evaluate and guide the translation of evidence-based interventions into real-world practice.

**Figure 1:**
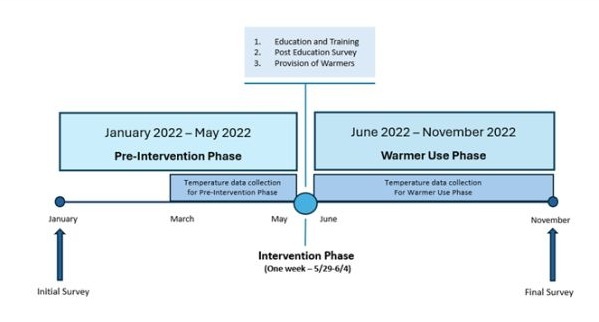
Study timeline.

The study consisted of three phases (**Figure 1**):

1) Pre-Intervention Phase: we administered an initial survey to health care providers, specifically nurses, physicians, and members of the transport team from the hospital and health centres. Then we collected Pre-Intervention data for three months.

2) Intervention Phase: we conducted an educational programme for the same health care providers, administered a Post-Education Survey, and distributed a total of 25 Infant Warmers to the hospital and its ten referral health centres.

3) Warmer Use (Post-Intervention) Phase: health care providers used the Infant Warmers at the hospital, health centres, and on transport for six months. We collected Post-Intervention data and administered a Final Survey at the conclusion of the study to assess the intervention's effectiveness.

### Data collection

We collected clinical data on all newborns admitted to the hospital during the Pre-Intervention and Warmer Use Phases. Our primary outcome measure was the rate of neonatal hypothermia before and after the intervention We collected temperature data at two-time points:

1) on admission, reflecting delivery room and transport management

2) six hours later, called ‘Follow-Up’ temperature, assessing the in-hospital management of hypothermia. We defined hypothermia as a temperature <36.5°C and fever as >37.5°C. Our demographic data was gestational age, birth weight, and sex.

To conduct our analysis, we used descriptive statistics with the assistance of Stata, BE 17.0 (StataCorp LLC, College Station, Texas, USA) to calculate the primary outcome. We used basic statistical metrics to evaluate the newborns' mean body temperature and hypothermia rates. To assess the differences in temperature between admission and Follow-Up, we employed paired *t* tests. Additionally, to determine the change in temperature from admission to Follow-Up between the Pre-Intervention and Warmer Use Phases, we conducted independent *t* tests.

### Surveys

We administered three surveys to health care providers at the hospital and health centres in which we assessed their knowledge of hypothermia and their confidence in caring for newborns and keeping them warm. The Initial Survey assessed baseline knowledge of neonatal hypothermia, identified gaps in the management of the heat chain of newborns after delivery, and determined areas for improvement in neonatal care. The Post-Education Survey evaluated the impact of the educational sessions. The Final Survey was conducted at the end of the Warmer Use Phase and assessed the long-term effect of the intervention on health care providers’ knowledge and practices, sustainability and acceptance, and perceived effect on mothers including KMC, breastfeeding, and bonding. The number of surveys were insufficient to perform formal qualitative research methodologies.

The surveys used a combination of Likert scale, multiple-choice, and free-text questions. All three surveys had four questions in common regarding overall thermoregulatory knowledge and management. The Post-Education Survey had additional questions regarding the training sessions. The Final Survey had additional questions regarding the perceived effectiveness, safety, and usability of the Warmer, and its impact on KMC, breastfeeding, and bonding.

Additionally, we conducted a Parent Survey of mothers whose newborns used the Dream Warmer. In this survey, we asked questions regarding the perception of the Warmer from a parent’s perspective, including its effect on KMC, breastfeeding, and bonding.

## RESULTS

We studied 372 newborns: 115 during the Pre-Intervention Phase and 257 during the Warmer Use Phase. The Pre-Intervention Phase went from January 2022 to May 2022; the Initial Survey was conducted in January, but the Pre-Intervention data collection was delayed until March due difficulties in importation and customs clearance of the Dream Warmer during the COVID pandemic. We conducted the education and training over one week to accommodate all shifts and personnel. The Warmer Use Phase went from June 2022 through November 2022 (**Figure 1**).

The demographic characteristics of the study population were comparable between the Pre- Intervention and Warmer Use groups (**Table 1**). The patient population was primarily Average for Gestational Age (AGA) term newborns, as expected in this low-risk community delivery hospital. We did not collect data on admission diagnosis.

**Table 1:** Demographic characteristics

Variables	Pre-Intervention Phase (n = 115)	Warmer Use Phase (n = 257)
Sex		
*Female*	56 (49%)	131 (51%)
*Male*	59 (51%)	126 (49%)
Gestational age (x̄)	38.7 weeks	38.4 weeks
Birthweight (x̄)	3219 grammes	3193 grammes

x̄ – mean

**Figure 2:**
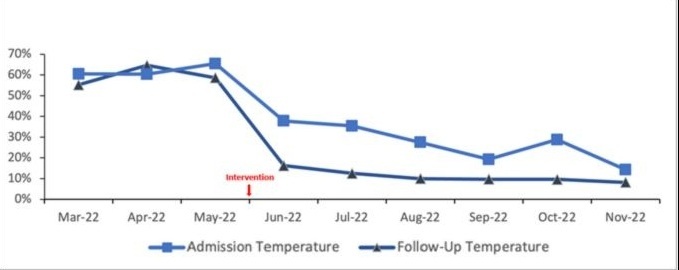
Hypothermia Rates Pre and Post Intervention.

During the Pre-Intervention Phase, the overall rate of hypothermia was 62% on admission, and 59% on Follow-Up. During the Warmer Use Phase, the rate of hypothermia decreased to 27% on admission, with a further decrease to 11% at Follow-Up (**Figure 2**). We do not have patient specific data on Warmer use to compare temperature of those who did and did not use the Warmer.

**Figure 3:**
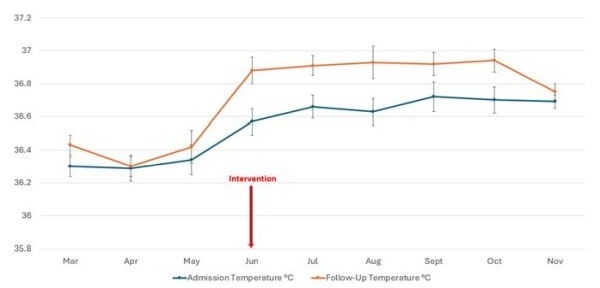
Temperatures Pre and Post Intervention.

The mean admission temperature increased (**Figure 3**, **Table 2**) from 36.31°C Pre-Intervention to 36.66°C during Warmer Use (*P* = 0.05). The mean Follow-Up temperature also increased from 36.37°C Pre-Intervention to 36.89°C during Warmer Use (*P* < 0.001). Thus, newborns’ mean Follow-Up temperature rose significantly compared to their admission temperature, with a mean increase from 0.06°C Pre- Intervention to 0.23°C during Warmer Use (*P* = 0.003). The incidence of fever was 3.4% Pre-Intervention and 3.1% during Warmer Use (*P* = 0.1) (**Table 2**).

**Table 2:** Effect of the intervention on temperature (°C)

Variables	n	Admission temperature, x̄ (SD)	Follow up temperature, x̄ (SD)	Change from admission	*P*-value	Difference between temperatures in the Pre-Intervention and Warmer Use Phases, x̄ (95% CI)	*P*-value	Fever
Pre-Intervention (March–May)	115	36.31 (0.47)	36.37 (0.45)	0.06 (0.37)	0.05	Reference		3.40%
Post-Intervention (June–November)	257	36.66 (0.49)	36.89 (0.47)	0.23 (0.50)	<0.001	0.16 (0.07, 0.25)	0.003	3.10%

CI – confidence interval, SD – standard deviation, x̄ – mean

Out of the 28 eligible health care providers, 27 completed the Initial Survey, 24 completed the Post-Education Survey, and 26 completed the Final Survey. Across the three surveys, health care providers knew the importance of keeping newborns warm. Yet, their knowledge of the normal temperature range, and their confidence in keeping newborns warm and providing overall newborn care rose over the course of the study (**Table 3**).

**Table 3:** Survey results of health care providers

Survey question*	Initial survey (n = 27)	Post-education survey (n = 24)	Final survey (n = 26)
Felt confident keeping newborns warm	8 (30%)	15 (62%)	23 (87%)
Perceived maintaining euthermia as very important	24 (92%)	22 (91%)	25 (96%)
Defined neonatal hypothermia correctly	10 (37%)	17 (70%)	22 (84%)
HCP is confident or very confident in overall newborn care	11 (40%)	7 (29%)	6 (23%)
Warmer is effective or very effective			23 (88%)†
Warmer positively influenced mother’s ability to provide KMC			21 (80%)†
Warmer had a positive effect on breastfeeding			20 (76%)†
Warmer promoted or strongly promoted mother-infant bonding			18 (69%)†
Warmer perceived to be safe			21 (80%)†
Warmer perceived as important or very important to treat hypothermia			26 (100%)
Warmer perceived to always be available when needed			22 (85%)

KMC – Kangaroo Mother Care, HCP – health care provider

*Free text questions: What did you like most about the Warmer? What did you like least about the Warmer? For summary of responses, see the results section.

†The rest of responses were neutral.

In the Final Survey, the Dream Warmer was reported to be safe, effective, and usable. Respondents reported that it promotes KMC, breastfeeding, and bonding. Health care providers described that they liked the Dream Warmer because it was ‘easy to use’ (n = 8), ‘practical’ (n = 8), ‘good for transport’ (n = 4), ‘efficient’ (n = 2), ‘safe’ (n = 1), ‘comfortable’ (n = 1), and ‘eased the workload’ (n = 1). Those who reported what they disliked about the Warmer cited the preparation time (n = 4), insufficient quantity of Warmers (n = 1), and the need to use water (n = 1).

Only five mothers answered the Parent Survey. They reported what they liked most about the Dream Warmer was that ‘my baby looked happy’, ‘(it) cured my baby from cold’, and ‘I like it’. Eighty percent reported that it promoted KMC, breastfeeding, and bonding. Mothers had no negative responses regarding the Warmer.

## DISCUSSION

Newborns, especially those who are small or sick, need a continuous external heat source to maintain euthermia, a basic condition for normal growth and physiological function. Our intervention successfully demonstrated the feasibility of addressing neonatal hypothermia in a low-resource setting where this issue was prevalent. By providing an appropriate thermoregulatory device and relevant education, we were able to reduce hypothermia rates by more than 2-fold at admission and 5-fold on Follow-Up and improve health care providers' knowledge and confidence in managing this condition. Mother’s perception of the Warmers was positive.

Despite the patient population primarily consisting of AGA term newborns, surprisingly many exhibited hypothermia on admission and during Follow-Up in the Pre-Intervention Phase. This finding demonstrates that hypothermia is not exclusively a concern for small and preterm newborns in cold climates, but also a significant issue for term newborns in a warmer climate. We note that the Pre-Intervention Phase occurred in the spring, and the Warmer Use Phase occurred during the late summer to fall. Because of the site’s proximity to the equator, there is relatively little minimum and maximum temperature variation across these seasons, though rainfall and humidity were higher in the Warmer Use Phase (Table S1 in the **Online Supplementary Document**). Therefore, we expect that the decrease in hypothermia rates during the Warmer use Phase was minimally affected by the change in seasons. Although we lack data on the frequency of Warmer usage, the substantial decrease in hypothermia rates observed during the study period suggests a successful adoption and implementation of this new device within the health care setting. We attribute this to the commitment of the study staff, who served as local advocates to encourage a culture shift towards recognising and addressing the pressing issue of hypothermia in newborns. Of note, when comparing the Pre-Intervention and Warmer Use Phases, the recorded temperature did not show an increase in rates of fever.

Studies have shown that health care providers in under-resourced settings often lack the equipment and knowledge to address hypothermia, leading to apathy and inadequate management of this prevalent condition [29,30]. Our outcome data and survey results show a sustained improvement in both rates of neonatal hypothermia and attitudes towards preventing it. One nurse surveyed said: ‘The Warmer gives us a heat source that we urgently need for babies in deliveries and is great for transport.’ These results demonstrate the power of implementation science to convert the fatalistic assumption that all small newborns will suffer, towards an increased awareness and demand for high quality inpatient care.

The open text answers by the health care providers gave us important information regarding the specific issue of preparing the Dream Warmer. In a previous qualitative research study [19] health care providers expressed concern about the potential for delay in Warmer use based on preparation time. We built this concern into our teaching material, emphasising the importance of preparing the Warmer in advance of need. We were pleased to note that 85% of health care providers describe the Warmers as being prepared in advance, and only 15% answered that what they like least about the Warmer was the preparation time.

The findings of our intervention exhibited strong results across all the RE-AIM components, highlighting the success of our intervention in addressing neonatal hypothermia in the targeted community and its potential for broader application in similar settings. Our intervention achieved the desired Reach, encompassing newborns at the community hospital of Jaltenango, at the ten community health centres, and on transport, and engaging a large proportion of eligible health care providers. The intervention Effectively reduced neonatal hypothermia rates and increased health care providers knowledge and comfort in treating the condition. High Adoption in the community was evidenced by survey results showing a favorable reception of the device by health care providers. Successful Implementation was demonstrated by the report of high usage of the warmer and the associated decrease in hypothermia rates, suggesting proper integration of both the device and the training into clinical practice. The sustained temperature data and survey responses throughout the study period indicate that the intervention's effects were Maintained over time, suggesting lasting benefits and potential integration into standard clinical practice.

Our study has several limitations. Although we provided education regarding the importance of euthermia, instruction on how to provide high quality KMC, and use of the Dream Warmer, we cannot distinguish to what degree each of these components contributed to the reduction in hypothermia rates due to limitations in our data collection. Because the research team provided education and distributed the Warmers, there may have been an observer effect where health care providers altered their behaviour due to awareness of being studied. The lack of patient-specific data on Warmer use makes it difficult to attribute temperature improvements solely to the intervention. Additionally, our data did not differentiate between inborn and transferred newborns, or if the Warmer was used in the health center, transport, or hospital setting. Therefore, we cannot characterise the effect of the Warmer in each of these settings. We only conducted five Parent Surveys due to time limitations of local study staff. Given the importance of family centered care, future studies should ensure additional data regarding parental perception. The relatively small number of Healthcare Provider and Parent Surveys was insufficient to conduct formal qualitative research methodologies.

## CONCLUSIONS

Given the importance of euthermia for survival with normal growth and development, scaling effective interventions aimed at combatting hypothermia are critical to optimising neonatal outcomes. Rates of hypothermia can be meaningfully reduced in a sustained manner with provision of appropriate education and equipment. Implementation Science gave us a practical means to provide and assess an evidence-based intervention in a real-world setting. This may serve as a replicable model for reducing neonatal hypothermia in similar settings. Further studies focusing on use-data in new geographical and cultural settings will provide valuable information to further refine the design of the Dream Warmer and the accompanying educational material. With hypothermia estimated to contribute to 40% of the 2.5 million newborns who die annually in low- and middle-income settings [31], addressing this preventable cause of mortality aligns with achieving to Neonatal 2030 Sustainable Development Goal of <12 deaths/1000 live births.

## Additional material


Online Supplementary Document
