# Supplementary material for: Prevention and treatment of neonatal hypothermia through an implementation science study in Jaltenango Chiapas, Mexico
Source: J Glob Health. 2025 Jun 2;15:04180. doi: 10.7189/jogh.15.04180 (PMC12127833; doi:10.7189/jogh.15.04180)
Supplement: Online Supplementary Document [file jogh-15-04180-s001.pdf]

Supplement to: Garcia AF, Azcarraga LS, Olivas MAE, Ulloa LZG, Santisteban RG, Hansen A. Prevention and treatment of neonatal hypothermia through an implementation science study in Jaltenango Chiapas, Mexico. J Glob Health. 2025;15:04180.

Table S1. Weather by Month in Jaltenango de la Paz

Month	Jan	Feb	Mar	Apr	May	Jun	Jul	Aug	Sep	Nov	Oct	Dec	Year
Record high °C (°F)	35.51 (95.92)	36.53 (97.75)	39.57 (103.23)	40.59 (105.06)	40.59 (105.06)	37.54 (99.57)	36.53 (97.75)	36.53 (97.75)	35.51 (95.92)	33.49 (92.28)	32.47 (90.45)	33.49 (92.28)	40.59 (105.06)
Average high °C (°F)	26.79 (80.22)	29.78 (85.6)	31.91 (89.44)	34.07 (93.33)	33.95 (93.11)	30.76 (87.37)	30.97 (87.75)	30.84 (87.51)	29.45 (85.01)	28.05 (82.49)	27.03 (80.65)	26.98 (80.56)	30.05 (86.09)
Daily mean °C (°F)	21.54 (70.77)	23.9 (75.02)	25.8 (78.44)	28.18 (82.72)	28.52 (83.34)	25.98 (78.76)	25.6 (78.08)	25.16 (77.29)	24.06 (75.31)	23.05 (73.49)	22.01 (71.62)	21.81 (71.26)	24.64 (76.35)
Average low °C (°F)	14.39 (57.9)	15.38 (59.68)	16.62 (61.92)	18.65 (65.57)	19.05 (66.29)	16.98 (62.56)	15.89 (60.6)	16.09 (60.96)	16.26 (61.27)	15.9 (60.62)	15.2 (59.36)	14.81 (58.66)	16.27 (61.29)
Record low °C (°F)	8.12 (46.62)	8.12 (46.62)	10.15 (50.27)	10.15 (50.27)	13.19 (55.74)	14.21 (57.58)	13.19 (55.74)	12.18 (53.92)	10.15 (50.27)	9.13 (48.43)	7.1 (44.78)	5.07 (41.13)	5.07 (41.13)
Average precipitation mm (inches)	55.78 (2.2)	37.17 (1.46)	54.58 (2.15)	75.79 (2.98)	174.39 (6.87)	433.88 (17.08)	352.99 (13.9)	496.8 (19.56)	457.38 (18.01)	300.72 (11.84)	96.39 (3.79)	42.85 (1.69)	214.89 (8.46)
Average precipitation days (≥ 1.0 mm)	10.89	9.41	10.89	14.76	22.97	28.87	27.85	29.52	29.24	25.46	14.95	8.03	19.4
Average relative humidity (%)	78.75	73.75	68.56	63.7	68.69	83.48	82.76	85.29	88.45	88.07	85.53	81.2	79.01
Mean monthly sunshine hours	9.96	11.5	11.45	12.44	13.02	12.78	12.81	12.55	11.19	8.43	8.35	8.49	11.08

Ref: https://weatherandclimate.com/mexico/chiapas/jaltenango-de-la-paz (last accessed 11/14/24)
